# Sub-chronic toxicity evaluation of top three commercial herbal antimalarial preparations in the Kumasi metropolis, Ghana

**DOI:** 10.1042/BSR20192536

**Published:** 2020-06-05

**Authors:** Frank Adusei-Mensah, Carina Tikkanen-Kaukanen, Jussi Kauhanen, Isaac Tabiri Henneh, Phyllis Elsie Owusu Agyei, Patrick Kafui Akakpo, Martins Ekor

**Affiliations:** 1 Institute of Public Health and Clinical Nutrition, School of Medicine, University of Eastern Finland, Kuopio campus, P.O. Box 1627 FI-70211, Kuopio, Finland; 2 School of Public Health and Social Work, Texila American University, George Town, Guyana; 3 Ruralia Institute, University of Helsinki, Lönnrotinkatu 7, 50100 Mikkeli, Finland and Helsinki Institute of Sustainability Science, University of Helsinki, Helsinki, Finland; 4 Department of Pharmacology, School of Medical Sciences, University of Cape Coast, P.M.B. University Post Office, Cape Coast, Ghana; 5 Department of Pathology, School of Medical Sciences, University of Cape Coast, P.M.B. University Post Office, Cape Coast, Ghana

**Keywords:** biochemical, congestion, haematology, haemolysis, histology, impaired, inflammation, safety, Surveillance

## Abstract

**Purpose:** Safety data on commonly used herbal medicinal (HM) products (HMPs) and marketed in Ghana are scarce. We assessed the sub-chronic toxicity of three most-patronised commercial antimalarial HMPs in Kumasi, Ghana.

**Method:** Top three HMPs (designated as herbal products ‘A’ (HPA), ‘B’ (HPB) and ‘C’ (HPC)) were selected after a mini-survey and sub-chronic toxicity evaluation conducted in accordance with Organisation for Economic Co-operation and Development (OECD) 407 guidelines. Control rats received clean water while test groups received daily adult human dose (DAHD), 5× DAHD or 10× DAHD of either HPA, HPB or HPC for 30 days. Rats were killed on day 31 to obtain biochemical, haematology and histology samples for analysis. Data were analysed by one-way analysis of variance (ANOVA) and *post hoc* Tukey’s test.

**Results:** The three HMPs produced alterations in liver morphology predominantly characterised by prominent foci of fatty change with scattered hepatocytes containing intracytoplasmic fat globules and congested central veins and sinusoids. The lungs showed alveolar with evidence of inflammation and foci of epithelial sloughing. Alveolar spaces were also obscured by debris and inflammatory cells. HPA and HPC produced scattered intensely congested heart vessels while HPB(10) produced haemorrhage and amorphous exudates within the heart. All HMPs produced neither treatment-related deaths nor significant change in haematological and biochemical parameters, except for HPA and HPB which decreased (*P*<0.05) aspartate aminotransferase (AST) and HPB, which elevated (*P*<0.05) fasting blood glucose (FBG).

**Conclusion:** Data from the present study suggest the potential of the herbal products (HPs), HPA, HPB and HPC, to cause major organ-system dysfunction or damage. We advise cautious use of these products and recommend further safety evaluation in chronic toxicity models.

## Introduction

Malaria is endemic in Ghana and other Sub-Saharan African countries. It is a leading cause of morbidity and mortality among children under 5 years and pregnant women in the region [1]. In 2016, the African World Health Organization (WHO) region reported 194.4 million malaria cases (90% of the global sum) and 405000 malaria deaths (91% of the global sum) [2,3]. Due to the high incidence density of approximately five malaria infections per person per year for Sub-Saharan African [4], and the high malaria morbidity and mortality rate, antimalarial medications are of great public interest in the region. Because of high cost and poor access to Artemisinin-based Combination Therapy (ACT), the use of plant-based multiherbal preparations (MHPs) for treatment of malaria is common practice in the region [1,5]. Year-round availability, affordability and easy accessibility of plant-based medicines compared with pharmaceutical medicines encourage their use. In fact, over 70% of Sub-Saharan Africans resort to the use of herbal medicines for their primary health needs [6,7]. In our recent survey, we observed that most of the subjects used herbal medicines solely or in combination with orthodox drug for various health needs including preventive, curative and chronic disease management. The study participants demonstrated high level of trust in herbal medicines and believed that herbal medicines are better curative agents than pharmaceutical medicine [8].

Most herbal medicinal (HM) products (HMPs) have been reported to be effective in treating liver problems [9], circulatory and respiratory diseases [10], and malaria [11]. In addition, medicinal plants also offer an unlimited and valuable recipe for the discovery of novel drugs. The lead compounds of several well-known pharmaceutical drugs have been discovered from plants. Artemisinin used in treating *Plasmodium falciparum* malaria was derived from *Artemisia annua*. The memory enhancer drug, physostigmine, was first isolated from *Physostigma venenosum* that grows in West Africa. Similarly, the anticancer drug, docetaxel, was originally obtained from *Taxus* species.

Recent scientific reports show that many medicinal plants employed as alternative medicines have adverse toxic effects comparable with those of pharmaceutical drugs [12]. There have also been reports on potential mutagenic, carcinogenic [13] and hepatotoxic [14] effects of some previously studied medicinal plants. Although, the leaves of *Cleistanthus collinus* are believed to possess anticancer properties, they have also been shown to contain a known human poison capable of causing mortality rate of 20–60% [15,16]. There is also a growing concern regarding contamination and adulteration of over-the-counter HMPs [17]. Although, toxicity profile of some medicinal plants or their extracts have been documented in Ghana, data on pre-market and post-market safety and toxicity are practically unavailable for most antimalarial and other MHPs on the Ghanaian market [18]. Regular safety surveillance of these commercial products is also lacking thereby creating data gap and raising public health concerns [19]. In our previous studies, we identified commercially available multiherbal products from the Ghanaian market with high levels of heavy metals and banned pesticide [20,21]. Supplementary Table S1 provides a summary of the ethnomedicinal uses and adverse or toxic effects of some medicinal plants employed in Traditional African Medicine. In the present study, we conducted a sub-chronic toxicity evaluation for three of the multiherbal antimalarial products previously analysed for their residual pesticide and heavy metal contents.

## Materials and methods

### Mini-survey and selection of herbal antimalarial products

In Ghana, consumers of HMPs do not need a doctor’s prescription to buy herbal medicine. The herbal medicine consumers (both patients and healthy individuals) buy their herbal products (HPs) from several sources including herbal centers, pharmacy shops and from those who sell at the roadsides, market places and to travellers in buses or at bus stations. In our previous study, a mini-survey was conducted to determine the most patronised antimalarial, antidiabetic and antihypertensive HPs on the Kumasi market [20,]. From the survey the top-three most patronised multiherbal antimalarial products, ‘Time Herbal Mixture®’, ‘Taabea Herbal Hixture®’ and Adutwumwaa Malamix® were then selected for further study (Supplementary Figure S1) [20,].

### Study design

The study design and different stages of the study are presented in Supplementary Figure S2. Stages of the study included HP selection, randomisation, grouping of the rats and the various studies performed on the rats.

### Animals and treatment schedule

Forty young healthy adult male Sprague–Dawley rats (10–12 weeks old) were used for the study. The animals were marked to permit easy identification and data handling and randomly assigned to ten groups of four rats per group (*n*=4). Each group was assigned to a cage and all the rats were kept for 10 days to allow acclimatisation to the laboratory conditions prior to dosing. Animals were maintained at ambient temperature and humidity with a 12-h light/12-h dark schedule and fed with standard pelleted rodent feeds and water *ad libitum* during the acclimatisation and experimental periods. Each of the nine groups of rats was assigned to one herbal antimalarial dose level and the tenth group that received clean water served as control. The animals were weighed every week prior to dosing with HPs and before they were killed under light chloroform anaesthesia on day 30.

The three commercial herbal products (HPs) selected for the study were assigned codes ‘HPA’, ‘HPB’ and ‘HPC’. The equivalent of the recommended daily adult human dose (DAHD) as stated on the product labels [marked as HPA(1), HPB(1) and HPC(1)] was used as the minimum dose and administered to rats. Five and ten times the DAHD were regarded as middle and highest doses respectively, and were marked as HPA(5), HPB(5), HPC(5) and HPA(10), HPB(10) and HPC(10), respectively (Supplementary Figure S1, Supplementary Tables S2 and S3). HPs and sterile water were administered daily for 30 days via the oral route. Housing, feeding, dosing of animals and daily observations were carried out as described in the Organisation for Economic Co-operation and Development’s (OECD 407) guideline. The number of animals assigned per group was in line with the 3R principle (reduce, refine and replace the use of animals). Care was also taken to avoid inflicting suffering and pain on the test animals in line with international principles and standards. The Animal Research: Reporting of *In Vivo* Experiments (ARRIVE) guidelines as well as the declaration of Helsinki (revised in 2013) were also observed.

### General observations

Once everyday after dosing and during the entire study period, the rats were observed for signs of toxic effects of the test products. The observations include rats' feeding, fur colour, self-isolation, signs of pains and death.

### Assessment of haematological parameters

Blood samples for haematology were collected into tubes containing sodium ethylenediaminetetraacetic acid (EDTA) (1% sodium EDTA in distilled water). Red blood cell (RBC), white blood cell (WBC), granulocyte (GRA), lymphocyte (LYM) count, haemoglobin (HGB), haematocrit (HCT), mean corpuscular haemoglobin (MCH), mean corpuscular volume (MCV), MCH concentration (MCHC), platelet (PLT) count, platelet distribution width (PDW), mean platelet volume (MPV) and platelet larger cell ratio (P-LCR) were estimated using haem automated analyzer (Cell Dyne: Model 331430, Abbott Laboratories, IL, U.S.A.).

### Biochemical analysis

Rats were weighed 12 h prior to euthanasia with chloroform and blood samples were collected via cardiac puncture. Five millilitres of blood was collected into gel separator sample tubes, allowed to clot and centrifuged at 3000 rpm for 15 min. The serum samples were separated, stored at −20°C and used for determination of biochemical parameters using automated biochemistry analyser (ATAC 8000, Elan Diagnostics, CA, U.S.A.). Biochemical parameters determined were bilirubin, alanine aminotransferase (ALT), aspartate aminotransferase (AST), alkaline phosphatase (ALP), total protein (TP), albumin (ALB), globulin (GLO), blood urea nitrogen (BUN), creatinine (CREA), glucose (GLU), low-density lipoprotein cholesterol (LDL-C), very LDL-C (VLDL-C), high-density lipoprotein cholesterol (HDL-C), triglyceride (TG) and total cholesterol (CHOL).

### Sperm count and fertility analysis

The analysis was carried out following standard procedures as described by Sreedhar and colleagues [22]. Briefly, small portion of cauda epididymis was cut and crushed in 1 ml of 37°C neutral buffer solution of sodium carbonate (NaHCO_3_) to make a homogeneous mixture. Then, 2–3 drops of Nigrosine stain were added into the mixture and 10 μl of the homogenate sample was pipetted on a pre-warmed slide. A minimum of ten fields were observed to evaluate the sperm count under the high-power light microscope at 40× magnification.

### Histology

Major vital organs (liver, heart, kidney, spleen, lungs and testis) were isolated, weighed and fixed in 10% v/v neutral buffered formalin and processed for histology. Briefly, small portions of each of the organs was carefully excised, dehydrated in graded alcohol and embedded in paraffin. Sections (4–10 μm thick) were prepared, stained with Haematoxylin and Eosin and mounted with neutral DPX medium. Examination of the slides was done using a light microscope (×40, ×100 and ×400).

### Data analysis

Results were analysed using one-tail analysis of variance (ANOVA) at 95% confidence interval. Tukey’s post hoc test was carried out on the data using SPSS version 21. GraphPad Prism version 8.0 was used for the graphical analysis and data were presented in charts and tables.

## Results

### Macroscopic assessment

Regular assessment of general drinking, feeding, appearance and exploratory behaviours (rearing and grooming) during toxicological investigations are important to detect toxicity-related behavioural changes that may be associated with the studied bioactive substance(s).

In the present study, general observation from macroscopic assessment of rats during the study did not show any adverse treatment-related changes in their feeding, exploratory behaviours and drinking habits. Incisor heights, fur colour and appearance were also normal.

### Body weight

Administration of certain xenobiotic substances may interfere with the normal feeding and drinking, disruption of gastrointestinal system and hormonal or enzymatic system interference. Feeding deterrents tend to decrease food intake and may lead to growth retardation or loss in body weight. In the present study we monitored the weights of the rats to assess the effect of the HPs on feeding and normal growth.

**Figure 1:**
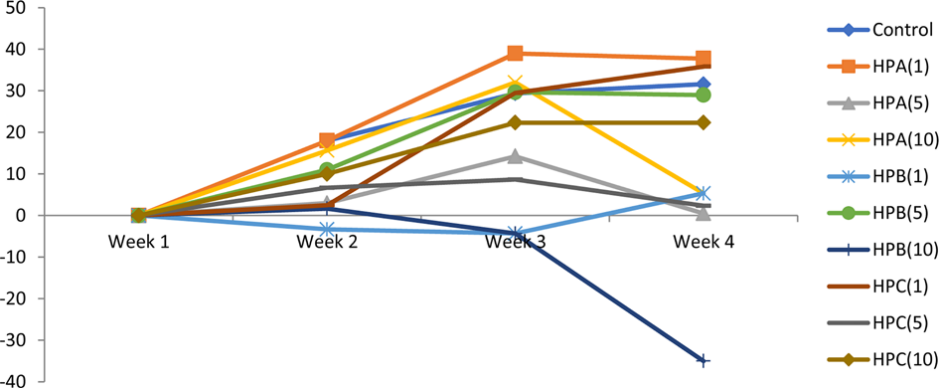
Weekly changes in body weight Values are expressed as mean ± SEM.

Generally, we observed weekly increases in weights across the groups for the first 3 weeks (Figure 1). Weight loss was observed across most of the groups in the fourth week except for the control and the first dose levels of both HPB and HPC. Continual weight loss was also observed in the highest dose group of HPB throughout the study. There were no statistical differences between the control and the compared groups.

### Parameters of haematological function

Therapeutic substances may produce adverse effects that may interfere with immunological process, inhibiting the action of important hormones, enzymes and by means of interfering with normal haematopoiesis [23]. Bioactive substances may inversely influence haematopoiesis resulting in different forms of anaemia. The body also increases production of different forms of WBCs in response to xenobiotics as part of normal body’s defense mechanism [24,25]. Haematological parameters were, therefore, assessed in the present study to determine the possible toxic effects of the herbal preparations on haematological function.

An increase in WBC count from 9.67 ± 2.50 HPA(5) to 10.67 ± 1.24 HPA(10) was observed (Table 1). Substantial increase in mixed cell count (MXD) consisting of monocytes, eosinophils, basophils (%) was also observed from 13.83 ± 3.19 HPA(5) to 20.10 ± 6.93 HPA(10) and 11.83 ± 3.08 of HPB(5) to 17.13 ± 2.77 of HPB(10) (Table 2). WBC increased from 8.57 ± 1.85 in HPB(1) to 15.07 ± 2.67 in HPB(5). Substantial MXD (%) increase was also observed from 13.83 ± 3.19 HPA(5) to 20.10 ± 6.93 in the highest dosed of HPA (4699.80 mg/kg body weight). These increases, however, were statistically not significant when compared with the control group. The HPC did not have significant impact on the haematological parameters when compared with the control group (Table 3).

**Table 1:** Effect of HPA on haematological parameters

Parameter	Control	HPA(1)	HPA(5)	HPA(10)
WBC (×10^3^/μl)	9.74 ± 0.52	8.13 ± 1.31	9.67 ± 2.50	10.67 ± 1.24
RBC (×10^6^/μl)	7.81 ± 0.23	8.07 ± 0.40	7.42 ± 0.43	7.90 ± 0.67
HGB (g/dl)	14.08 ± 0.42	14.3 ± 0.67	12.67 ± 0.60	13.97 ± 0.57
HCT (%)	47.40 ± 1.34	47.63 ±2.06	41.83 ± 2.66	47.87 ± 1.94
MCV (fl)	60.72 ± 1.18	59.03 ± 0.50	56.33 ± 0.67	61 ± 2.60
MCH (pg)	18.08 ± 0.58	17.73 ± 0.12	17.10 ± 0.21	17.80 ± 0.78
MCHC (g/dl)	29.70 ± 0.45	30.00 ± 0.31	30.37 ± 0.46	29.20 ± 0.53
Platelets (×10^3^/μl)	1152 ± 58.95	883.3 ± 198.00	1060 ± 86.53	987.3 ± 99.55
Lymphocytes (%)	75.96 ± 3.11	77.53 ± 3.50	57.23 ± 15.14	63.37 ± 9.59
MXD (%)	15.02 ± 1.87	13.83 ± 3.19	20.10 ± 6.93	18.47 ± 10.10
Neutrophils (%)	9.02 ± 1.45	8.63 ± 0.58	22.67 ± 8.89	18.17 ± 10.58
LYM #(×10^3^)	7.34 ± 0.367	6.40 ± 1.33	5.37 ± 2.17	6.83 ± 1.33
MXD #(×10^3^)	1.48 ± 0.24	1.07 ± 0.12	1.933 ± 0.89	1.73 ± 0.81
NEUT #(×10^3^)	0.92 ± 0.16	0.67 ± 0.09	2.37 ± 1.13	2.10 ± 1.46
RDW_SD (fl)	39.02 ± 0.91	37.90 ± 1.07	35.10 ± 0.71	36.77 ± 2.09
RDW_CV (fl)	18.30 ± 0.44	18.23 ± 0.35	17.23 ± 0.87	16.50 ± 1.18
PDW (fl)	11.78 ± 0.17	13.43 ± 0.42	14.00 ± 2.30	11.77 ± 0.18
MPV (fl)	9.26 ± 0.11	10.07 ± 0.55	10.23 ± 0.94	9.43 ± 0.23
P-LCR (%)	20.18 ± 0.89	27.93 ± 4.33	28.40 ± 8.11	21.30 ± 1.42

Values are expressed as mean ± SEM. *P*-values less than 0.05 were considered statistically significant.

**Table 2:** Effect of HPB on haematological parameters

Parameter	Control	HPB(1)	HPB(5)	HPB(10)
WBC (×10^3^/μl)	9.74 ± 0.52	8.57 ± 1.85	15.07 ± 2.67	9.13 ± 2.23
RBC (×10^6^/μl)	7.81 ± 0.23	7.33 ± 0.48	8.28 ± 0.53	7.48 ± 0.50
HGB (g/dl)	14.08 ± 0.42	13.3 ± 0.63	14.43 ± 0.74	13.57 ± 0.85
HCT (%)	47.40 ± 1.34	44.77 ± 2.43	48.5 ± 2.96	44.43 ± 3.51
MCV (fl)	60.72 ± 1.18	61.17 ± 0.72	58.57 ± 0.27	59.3 ± 2.18
MCH (pg)	18.08 ± 0.58	18.20 ± 0.32	17.47 ± 0.42	18.17 ± 0.47
MCHC (g/dl)	29.70 ± 0.45	29.73 ± 0.23	29.80 ± 0.55	30.30 ± 0.85
Platelet (×10^3^/μl)	1152 ± 58.95	99.55 ± 44.06	1077 ± 117.60	1266 ± 260.60
Lymphocytes (%)	75.96 ± 3.11	81.40 ± 2.23	72.03 ± 2.72	67.87 ± 4.24
MXD (%)	15.02 ± 1.87	13.03 ± 1.93	11.83 ± 3.08	17.13 ± 2.77
Neutrophils (%)	9.02 ± 1.45	5.57 ± 1.04	16.13 ± 5.72	15 ± 3.12
LYM #(×10^3^)	7.34 ± 0.367	7.07 ± 1.68	10.73 ± 1.49	6.07 ± 1.35
MXD #(×10^3^)	1.48 ± 0.24	1.03 ± 0.13	1.63 ± 0.18	1.57 ± 0.48
NEUT #(×10^3^)	0.92 ± 0.16	0.47 ± 0.09	2.70 ± 1.38	1.50 ± 0.57
RDW_SD (fl)	39.02 ± 0.91	37.50 ± 1.76	36.73 ± 1.28	37.60 ± 2.17
RDW_CV (fl)	18.30 ± 0.44	16.33 ± 1.33	17.80 ± 1.03	17.17 ± 0.09
PDW (fl)	11.78 ± 0.17	12.40 ± 0.40	11.93 ± 0.75	13.00 ± 0.67
MPV (fl)	9.26 ± 0.11	9.80 ± 0.38	9.33 ± 0.27	10.13 ± 0.37
P-LCR (%)	20.18 ± 0.89	23.83 ± 2.83	20.57 ± 2.64	27.13 ± 2.98

Values are expressed as mean ± SEM. *P*-values less than 0.05 were considered statistically significant.

**Table 3:** Effect of HPC on haematological parameters

Parameter	Control	HPC(1)	HPC(5)	HPC(10)
WBC (×10^3^/μl)	9.74 ± 0.52	11.10 ± 1.04	11.77 ± 0.45	7.13 ± 2.45
RBC (×10^6^/μl)	7.81 ± 0.23	8.01 ± 0.16	7.73 ± 0.53	8.06 ± 0.38
HGB (g/dl)	14.08 ± 0.42	14.43 ± 0.54	13.97 ± 0.56	14 ± 0.17
HCT (%)	47.40 ± 1.34	47.77 ± 1.67	45.03 ± 1.65	46.8 ± 0.60
MCV (fl)	60.72 ± 1.18	59.6 ± 1.07	58.5 ± 2.34	58.3 ± 2.91
MCH (pg)	18.08 ± 0.58	18.03 ± 0.41	18.13 ± 0.64	17.43 ± 0.73
MCHC (g/dl)	29.70 ± 0.45	30.20 ± 0.15	31.00 ± 0.17	29.93 ± 0.77
Platelet (×10^3^/μl)	1152 ± 58.95	1135 ± 144.60	1058 ± 80.71	1020 ± 57.30
Lymphocytes (%)	75.96 ± 3.11	71.93 ± 4.04	65.80 ± 6.05	72.53 ± 9.12
MXD (%)	15.02 ± 1.87	16.80 ± 2.33	21.67 ± 3.58	11.57 ± 2.39
Neutrophils (%)	9.02 ± 1.45	11.27 ± 1.73	12.53 ± 2.48	15.90 ± 6.91
LYM #(×10^3^)	7.34 ± 0.367	8.00 ± 0.95	7.77 ± 0.87	4.77 ± 1.09
MXD #(×10^3^)	1.48 ± 0.24	1.83 ± 0.23	2.53 ± 0.41	0.90 ± 0.50
NEUT #(×10^3^)	0.92 ± 0.16	1.27 ± 0.20	1.47 ± 0.29	1.47 ± 0.97
RDW_SD (fl)	39.02 ± 0.91	38.50 ± 0.71	35.73 ± 1.43	35.70 ± 2.85
RDW_CV (fl)	18.30 ± 0.44	18.23 ± 0.47	16.73 ± 0.73	17.00 ± 0.76
PDW (fl)	11.78 ± 0.17	12.57 ± 0.32	12.70 ± 0.66	11.67 ± 0.37
MPV (fl)	9.26 ± 0.11	9.87 ± 0.30	10 ± 0.45	9.63 ± 0.19
P-LCR (%)	20.18 ± 0.89	25.27 ± 2.04	26.40 ± 4.25	22.13 ± 2.03

Values are expressed as mean ± SEM. *P*-values less than 0.05 were considered statistically significant.

### Parameters of biochemical function

AST is primarily found in the cytoplasm and mitochondria of cardiac muscle, liver and skeletal muscle [26]. ALT, on the other hand, is found primarily in the cytosol of hepatic cells. During necrosis, liver injury or alteration in hepatocellular permeability, liver enzymes leak into the bloodstream and serum levels of these enzymes, therefore, serve as good markers for assessment of hepatotoxicity. AST and the specific liver enzyme, ALT, are used to assess the integrity of the hepatic cells since they give indication of the degree of hepatocytes degeneration [12]. Indirect bilirubin is formed from the breakdown of RBC’s HGB. It is conjugated to glucuronic acid in the liver (direct bilirubin) and excreted via bile. Bilirubin test is important to access degree of RBC haemolysis and the catabolic function of the liver during bioactive toxicity studies. The kidney plays important role in excreting waste substances including bilirubin and urea from the body. In addition, the liver also plays a crucial role in protein and lipid biosynthesis in the body. Approximately 80% of cholesterol used in the mammalian body is endogenously made. Most cells of the body synthesise cholesterol for their own usage while the liver’s cholesterol is for transportation and other purposes. Most serum endogenous cholesterol therefore has the liver as its origin and their levels may, among other things, tell about the synthetic ability of the liver after drug administration.

**Figure 2:**
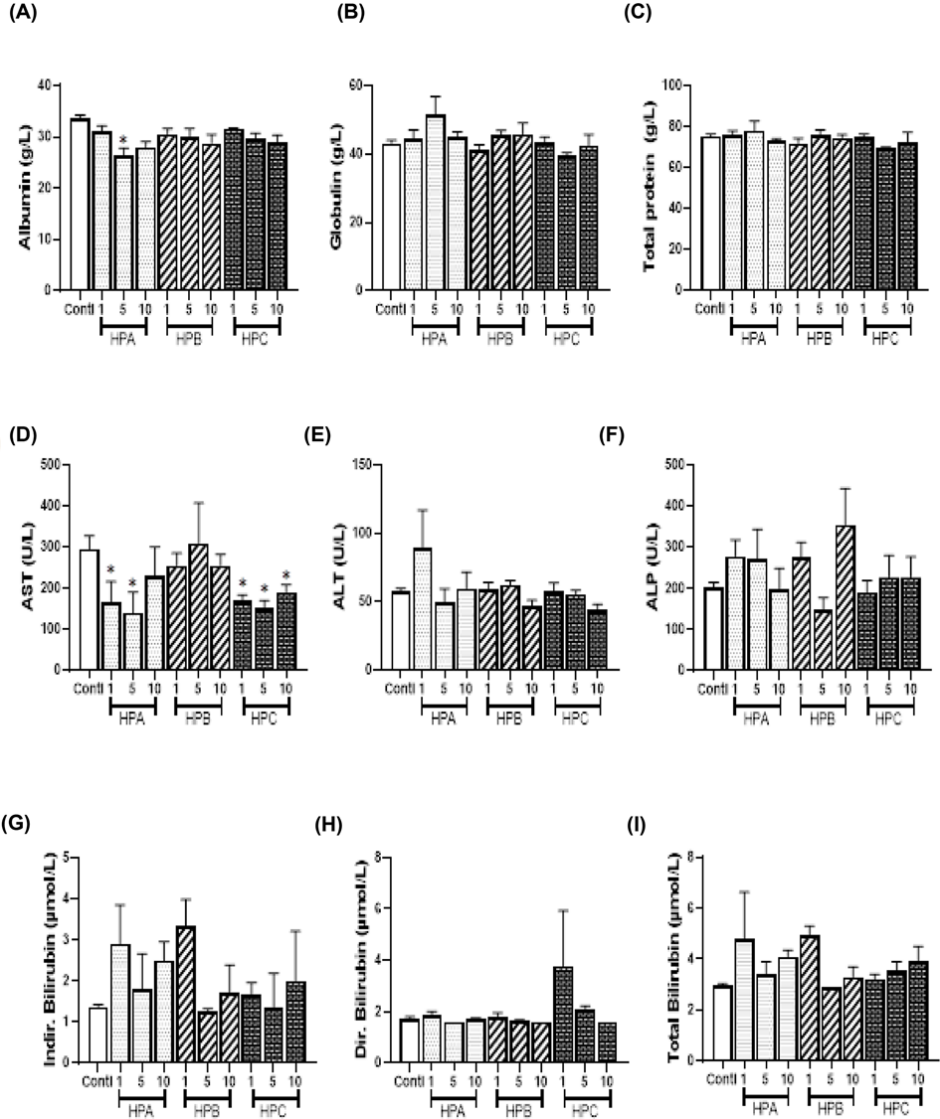
Serum activities of liver enzymes and levels of proteins and bilirubin Values are expressed as mean ± SEM. Statistical difference between the test group and the control at 95% CI is marked with * at *P*-values less than 0.05. (**A-C**): levels of serum proteins, (**D-F**): serum activities of liver enzymes, (**G-I**): serum bilirubin concentrations.

**Figure 3:**
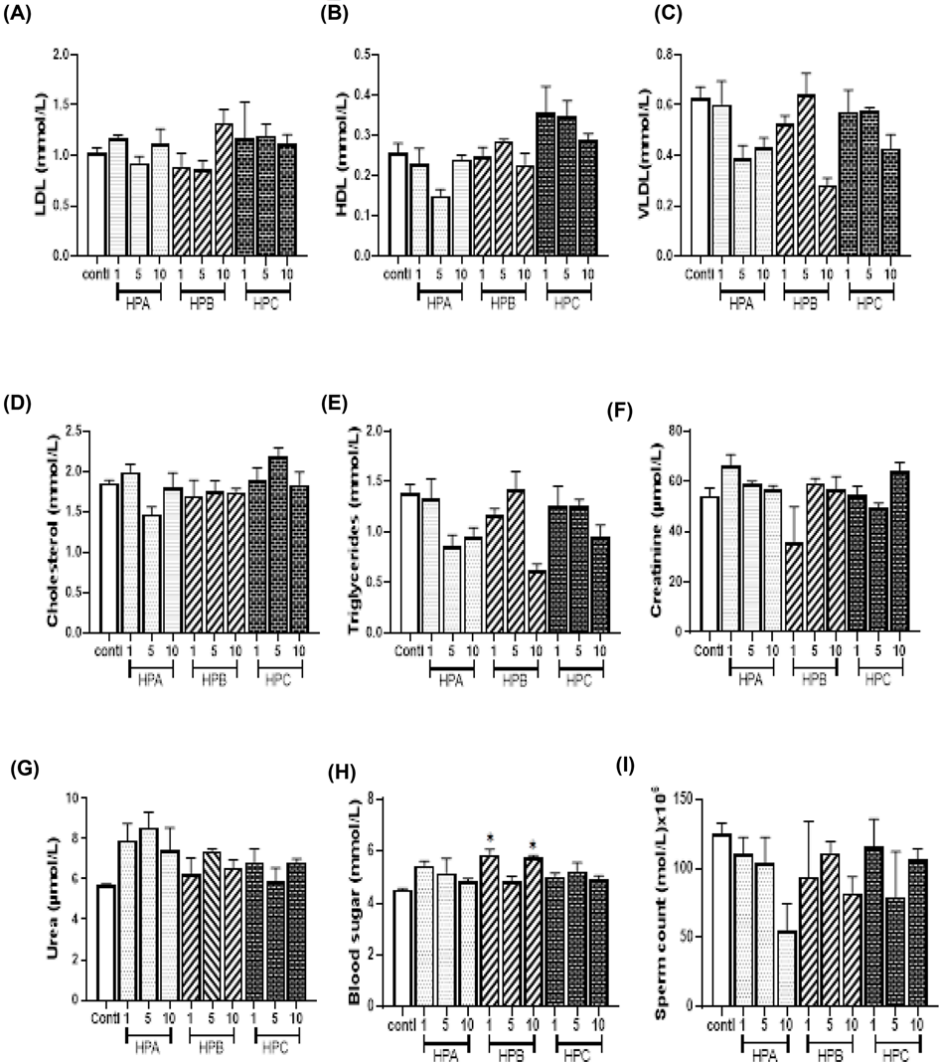
Lipid profile, serum creatinine, blood urea nitrogen, blood sugar and sperm count Values are expressed as mean ± SEM. Statistical difference between the test group and the control at 95% CI is marked with * at *P*-values less than 0.05. (**A-E**): serum lipid profile, (**F**): serum creatinine concentration, (**G**): blood urea nitrogen, (**H**): blood sugar concentration, (**I**): sperm count.

Creatinine (the waste product from the normal wear and tear on muscles of the body) and blood urea nitrogen (byproduct of protein metabolism) are cleared regularly by the kidney [27]. The levels of serum creatinine and blood urea nitrogen are therefore useful indicators of kidney function. We observed that indirect bilirubin levels increased with dose for HPA but decreased with increasing doses of HPB. Albumin, total and direct bilirubin levels also decreased with increasing doses for HPA. A significantly low AST activity was observed in the middle-dose level of HPA and at all dose levels of HPC when compared with the control group (Figure 2). The effects of HPA and HPB on AST and ALT activities were not dose related. Albumin level was also significantly low in HPB(5) dose group (Figure 2). The least, middle and the highest dose groups of AST were all significantly low when compared with the control group (Figure 2). LDL-C level non-significantly increased at the dose of HPB(10) while VLDL-C level substantially reduced for HPB(10) group. Reduced HDL-C and CHOL levels were also observed for HPB(5). Fasting blood sugar levels for HPB(1) and HPB(10) were significantly higher than in the control group (Figure 3). Most of the lipid parameters for rats in the HPC groups were not significantly different from those of the control group (Figure 3). Similarly, serum creatinine and blood urea nitrogen of all dose groups of HPA, HPB and HPC were not significantly different from the control group (Figure 3).

### Relative organ weight

Toxic effects exerted by certain bioactive substances are capable of inducing inflammatory responses in different tissues and organs leading to damage. Inflammation in essential organs may lead to increase in weight and higher organ-to-body weight ratios compared with the normal organs. Organ weights and organ-to-body weight ratio comparison between the treated groups and the control has conventionally been used to evaluate toxic effects of bioactive products. The Society of Toxicologic Pathology considers organ weight evaluation to be an important screening tool in characterising the toxicity of a bioactive substances in general toxicity studies [28]. In the present study the weights of the heart, liver, kidney, spleen and testis and their relative weights with regards to the body weights were evaluated.

**Figure 4:**
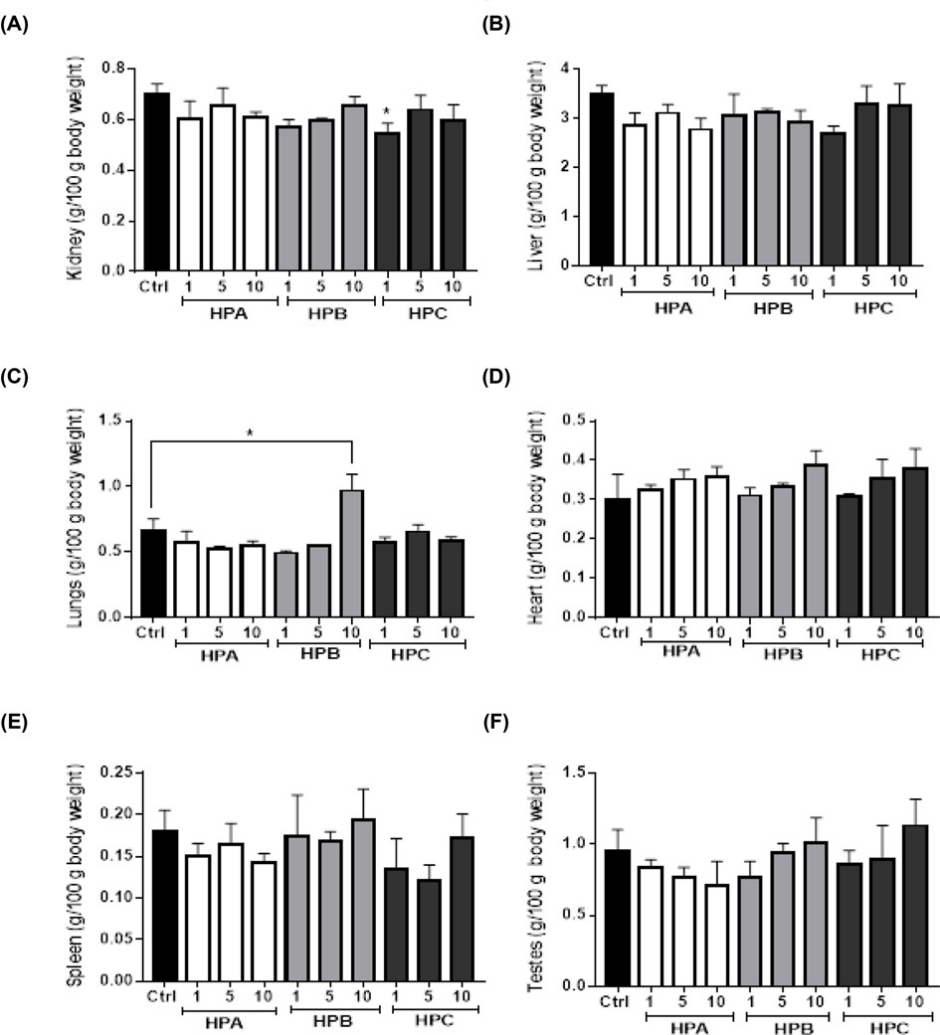
Relative organ weights per 100 g body weight Values are expressed as mean ± SEM. Statistical difference between the test group and the control at 95% CI is marked with *. Organ to body weight ratio (g/100g body weight) of (**A**): kidney, (**B**): liver, (**C**): lung, (**D**): heart, (**E**): spleen, (**F**): testes.

The relative kidney weight of rats in the least dose group of HPC (774.00 mg/kg body weight) was significantly lower than those of the control group. Similarly, the relative lung weight of rats in the highest dose group of HPB (3999.60 mg/kg body weight) was significantly higher when compared with the control group (Figure 4). However, the weights of the heart, the liver, the spleen and the testis of the test groups were not significantly different from the control group.

### Histologic assessment

Analysis of the microscopic structure of essential tissues and organs is often recommended in toxicity or safety assessment of bioactive substances [29]. Such histological evaluation is crucial in detecting presymptomatic toxic effects that may not be observed in biochemical analysis. In the present study, we examined the microscopic structures of the heart, lung, liver, kidney, spleen and testis of the experimental animals after exposure to the HMPs. We also examined these organs in the control group to allow for comparison.

Infertility among married couples is on the increase in Ghana [30,31] and finding the cause is a concern to all public health practitioners. Certain bioactive substances impair fertility by adversely affecting spermatogenesis. We therefore, assessed sperm quality and quantity in the present study in order to determine the impact of the herbal preparations on fertility of the male rats.

#### Liver

**Figure 5:**
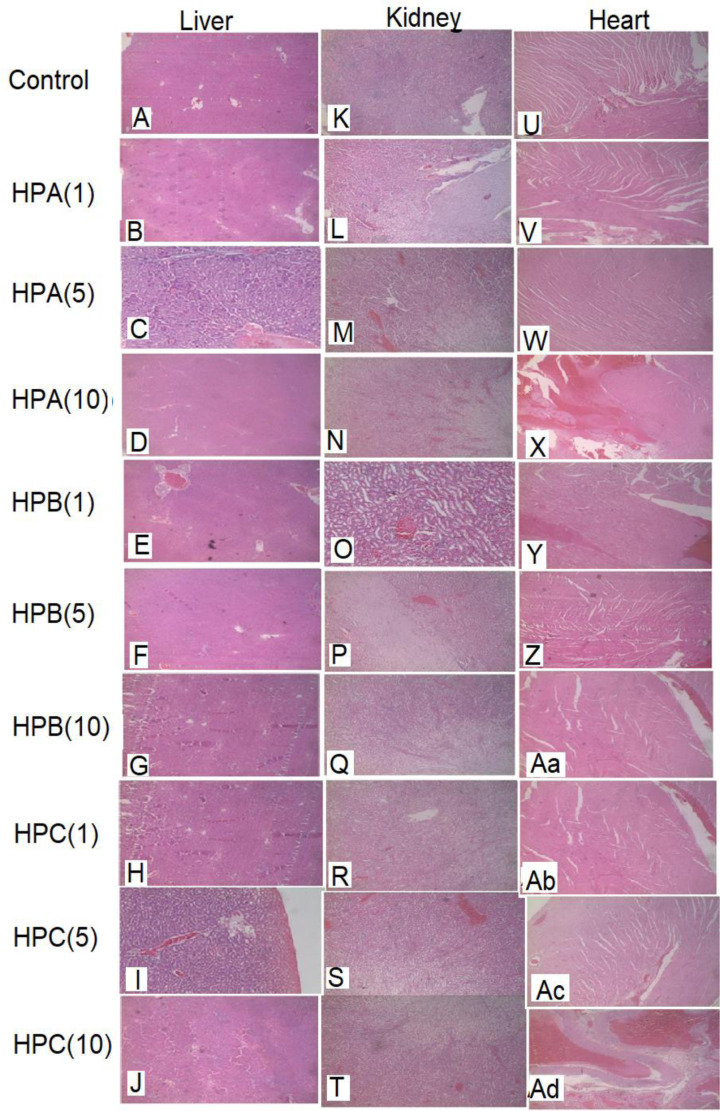
Photomicrographs of the liver, kidney and heart for the sub-chronic toxicity studies of Sprague–Dawley rats treated with either sterile water, HPA, HPB or HPC for 30 days Magnification, ×100. Haematoxylin–Eosin stain was used. Photomicrographs of sections of (**A-J**): liver, (**K-T**): kidney, (**U-Z**) and (**Aa-Ad**): heart.

Liver sections of control rats revealed a moderate to severe level of congestion within the central veins (Figure 5A). Sections from the liver of rats that received HPA(1–10) exhibited congestion within the central veins, sinusoids, large vessels and under the capsule suggesting subcapsular haemorrhage (Figure 5B–D). In addition, foci of necrosis without evidence of inflammation were observed in HPA(1) dose group. Also, scattered individual hepatocytes that showed intracytoplasmic fat globules were seen in rats that received HPA(5) and HPA(10). The liver of rats given HPB(1) exhibited central vein congestion (Figure 5E). Also, liver section from rats administered with HPB(5) and HPB(10) showed normal architecture with congestion of central veins and sinusoids (Figure 5F,G). In addition, the hepatocytes of rats in the HPB(5) group revealed prominent foci of fatty changes with mainly microvesicular conformation. Rats in the HPC(1, 5,10) dose groups (Figure 5H–J) revealed liver sections with congestion within the central veins, sinusoids, large vessels and under the capsule. In addition, a focus of foamy hepatocytes was observed in rats treated with HPC(5).

#### Kidney

Control rats and the group that received HPB(1) showed normal renal architecture with normal glomeruli. Renal tubules and collecting ducts showed mild congestion within their stroma (Figure 5K,O). All HPA, HPB and HPC dose groups except HPB(1) showed renal tubules and collecting ducts with mild congestion within their stroma and within the glomeruli (Figure 5). In addition, there were foci of chronic inflammatory change in the highest dose group of HPC (Figure 5T).

#### Heart

Scattered intensely congested heart vessels, without evidence of inflammation, infarction or fibrosis were observed at all dose levels of HPA (Figure 5V–X) and HPC(1, 5, 10)-treated groups (Figure 5Ab–Ad). Intensely eosinophilic muscle fibres, haemorrhage with associated amorphous exudates in isolated muscle fibres as well as scattered intensely congested vessels were seen in the heart sections of rats that received HPB(10) (Figure 5Aa).

#### Lungs

**Figure 6:**
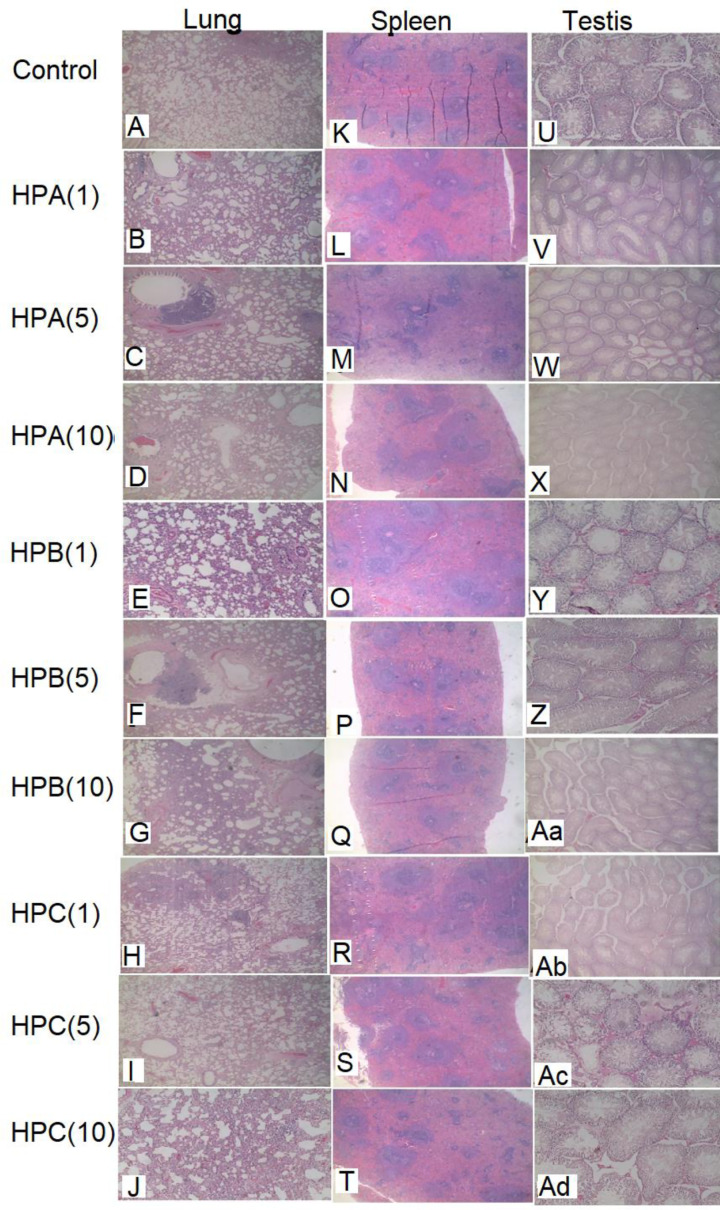
Photomicrographs of the lung, spleen and testis for the sub-chronic toxicity studies of Sprague–Dawley rats treated with either sterile water, HPA, HPB or HPC for 30 days Magnification, ×100. Heamatoxylin–Eosin stain was used. Photomicrographs of sections of (**A-J**): lung, (**K-T**): spleen, (**U-Z**) and (**Aa-Ad**): testis.

Lung sections of rats that received clean water showed foci of epithelial sloughing within the airway (Figure 6A). Evidence of chronic inflammation and groups of chronic inflammatory cells filling alveolar spaces and foci of sloughing within the airway were observed in HPA(1)-treated rats (Figure 6B). Rats in the HPA(5) group had lungs that showed chronic inflammatory changes dominated by lymphocytes and macrophages with sloughing of epithelial cells. The alveolar spaces were obscured by debris and chronic inflammatory cells (Figure 6C). Lungs of rats given HPA(10) also showed moderate chronic inflammatory changes dominated by lymphocytes and macrophages with sloughing of epithelial cells in some areas. The alveolar spaces are obscured by debris and chronic inflammatory cells (Figure 6D). Rats that received HPB(1) showed lung exhibiting thickened alveolar septae and chronic inflammatory cells with congestion within the lung parenchyma. There was also evidence of non-specific chronic inflammation (Figure 6E). Sections of lung from rats that received HPB(5) showed alveolar with evidence of chronic inflammation in some areas with groups of chronic inflammatory cells. There were foci of sloughing within the airway (Figure 6F). The lung section of rats treated with HPB(10) exhibited alveolar that showed little evidence of inflammation in some areas and foci of sloughing within the airway (Figure 6G). Alveolar with evidence of moderate chronic inflammation in many areas with groups of chronic inflammatory cells filling alveolar spaces were observed in lung section of rats that received HPC (Figure 6H–J). In addition, there were foci of sloughing observed within the airway of HPC(1, 5, 10)-treated groups.

#### Spleen

The HPs (HPA, HPB and HPC) did not affect the histology of the spleens of the rats when compared with the control group (Figure 6K–T).

#### Testis

Sections of testis from control rats and from rats that received different doses of HPA, HPB and HPC showed normal architecture with seminiferous tubules containing cells at all stages of spermatogenesis without any evidence of inflammation or adverse drug effect (Figure 6U–Ad).

## Discussion

HMPs are commonplace on the Ghanaian market and their use is on the increase due to the growing demand for nature-based products. In spite of the huge patronage and extensive use of HMPs for management of various ailments in Ghana, information regarding their safety is seldom available. The paucity of scientific data on the possible harmful effects of these multiherbal products prompted the present investigation in which we evaluated the safety of the three most patronised antimalarial HMPs in the Kumasi metropolis of Ghana in an experimental model of sub-chronic toxicity study. Data from the present study did not reveal any significant impairment of haematological function or treatment-related deaths in the various test groups. Red blood cell and WBC counts with differential analysis did not reveal any signs of macrocytic or microcytic anaemia in all groups that received the HPs. While slight elevation was observed in the RBC counts of some groups, the values were not significantly different from those of the control group and as such was considered toxicologically not important. Similarly, HPB (middle and high dose levels) and HPC (low and middle dose levels) increased WBC counts, although values were not statistically different from those of the control. Macroscopic examination of gastrointestinal tract after animals were killed and dissected revealed mild ulcerations in the small intestines of rats that received HPB (high dose).

Our results also showed that sub-chronic administration of HPA (at low and middle doses) and HPC (at all doses) significantly decrease AST in the rats. These findings suggest either a liver protective effect, chronic toxic effect or death of the liver cells due to extensive toxic effect of the HPs. It could also be a mixture of some of these effects since the products have been stated to comprise three to five different herbal plants with different pharmacological properties. The argument for a possible liver protective effect by HPC is partly supported by previous studies which found one of the active ingredient of HPC ‘*Phyllanthus fraternus*’ to possess antihepatotoxic activity [32,33], justifying its used in traditional medicine for the treatment of jaundice. This assertion on HPC also correlates well with a clinical trial of the same product which was published at the time this current study was ongoing [34]. On the other hand, the argument for a possible chronic liver toxicity induced by the HPs is supported by the previously identified chemical toxicants in these products [20,35]. Histologic findings from this study, however, support the induction of liver toxicity by the HMPs. For instance, congestion of sinusoids, within the large vessels and under the capsule were observed in all dose groups of HPA, HPC and HPB(5 and 10). There were foci of necrosis in HPA(1), hepatocytes with intracytoplasmic fat globules in HPA(5 and 10), hepatocytes with prominent foci of fatty change with mainly microvesicular conformation in HPB(5) group and congestion within the large vessels and under the capsule and a focus of foamy hepatocytes observed in HPC(5). In addition, the fat globules observed in HPA(5 and 10) affected the liver’s synthetic ability leading to a significantly low albumin level in HPA(5). The assertion was strengthened by the fact that the albumin/globulin ratio of HPA (5 and 10) group was much less when compared with control group. A component of the HPC ‘*Vitex grandifolia’* had previously been identified to be toxic to Sprague–Dawley rats and this conforms well with the toxic observations made in the present study.

The fasting blood sugar levels for rats treated with low and high doses of HPB (1 and 10) were significantly higher when compared with the control group. This hyperglycaemia may suggest possible interference with glucose metabolism or injury to the pancreas and a possible predisposition of the rats to diabetes mellitus. The association of hyperglycaemia and development of diabetes mellitus has been well studied [36]. By extension, since the least dose of HPB(1) (which is the equivalent dose recommended for human use) significantly increased blood glucose levels in the rats, it is likely that chronic exposure at this dose may predispose the consumer to the risk of diabetes mellitus.

Furthermore, rats administered with HMPs (at all dose levels) exhibited signs of moderate lung toxicity. This is evident from lung histology that revealed signs of pulmonary toxicity characterised by inflammation in all dose groups of HPA and HPB, in addition to congestion and presence of chronic inflammatory cells in rats that received HPC. In addition, the relative weight of the lungs of HPB(10) group was significantly higher than that of the control group. In our recent study, we identified HPs (A, B and C) to have been contaminated with nickel, lead and chromium (residual contents greater than maximum residual limit (MRL) values) [29], and previous studies have also linked Ni overexposure to lung injury, inflammation, fibrosis and cancer of the respiratory tract [37]. Considering the dose-related inflammatory changes in the rat lungs and other signs of pulmonary toxicity, it is possible, therefore, that exposure to the Ni content of the HPs may contribute to the observed lung toxicity.

Data from the present study further revealed that rats treated with HPB(10) also exhibited gastrointestinal lesions, especially in the small intestine. GIT lesions are typical signs of chromium (VI) toxicity [38,39]. The induction of GIT lesions by HPB seems to correlate with our recent study where we reported the presence of significantly high levels of Cr above MRL limit in this herbal preparation [20]. Similar morphological changes were observed in the lungs of rats that received HPC with the alveolar showing inflammatory changes dominated by lymphocytes and macrophages and alveolar spaces obscured by debris and chronic inflammatory cells which could be attributed to the heavy metal contaminations previously identified at concentrations above their respective MRL values [20,37].

We also observed signs of mild kidney toxicity in rats administered with HPA and HPB (at all dose levels) and moderate kidney toxicity in rats given HPC(1–10), characterised by congestion within the glomerular capillary and the stroma of the renal tubules and collecting ducts. Foci of chronic inflammation were also seen in rats that received HPC(10). In addition, the relative kidney weight of the rats that received HPC(1) was significantly lower than those of the control group. The chronic inflammation and possible occurrence of atrophy could explain the significantly low relative kidney weight in HPC(1) [40,41]. The observed foci of chronic inflammation in the present study may be related to the presence of heavy metals (arsenic, chromium and nickel) and pesticides (aldrin and dieldrin) as previously stated and reported in our recent article [20]. This also correlates with previous findings on chromium (VI), [38] arsenic [42], and aldrin [37] toxicities.

We observed that the HMPs (HPA, HPB and HPC), at the dose given and duration of administration in the present study, did not exert any adverse influence on sperm characteristics. This is evident in the similarity between sperm count and sperm morphology of test rats and those of the control group.

Sub-chronic administration of HPA(1-10) and HPC(1-10) to rats caused moderate myocardial toxicity marked by scattered but intensely congested vessels. The heart sections of rats given HPB(10) show foci of intensely eosinophilic muscle fibres, haemorrhage with associated amorphous exudates, isolated muscle fibres and scattered intensely congested vessels. This effect may in part be attributable to the high concentration of heavy metals like arsenic in these products [20]. Association of arsenic and inflammatory damage to the vascular system has been reported in literature. Inflammatory changes and vascular congestion are signs typically associated with arsenic heart toxicity [42,43]. Although the various medicinal plant components of the antimalarial HMPs investigated in this study may be linked to the toxic effects observed in the rats, we strongly believe that the heavy metal and pesticide contaminants found in these herbal preparations are also playing a major role.

## Conclusion

Data from the present study suggest the potential of the HPs, HPA, HPB and HPC, to cause major organ-system dysfunction or damage. The observed toxic effects of these HMPs may be related in part to their contamination with heavy metals and pesticides. The need for alternative, safe drugs for treatment of malaria is huge. We recommend the use of these HPs with caution and suggest further assessment of their safety in a chronic model of toxicity. We also recommend good manufacturing as well as safe farming practices to reduce contamination of herbal medicines with heavy metals, pesticides or other pollutants.

## Supplementary Material

Supplementary Figure S1-S2 and Tables S1-S4

## Abbreviations

ALP: alkaline phosphatase
ALT: alanine aminotransferase
AST: aspartate aminotransferase
CHOL: total cholesterol
DAHD: daily adult human dose
EDTA: ethylenediaminetetraacetic acid
FBG: fasting blood glucose
GRA: granulocyte
HCT: haematocrit
HDL-C: high-density lipoprotein cholesterol
HGB: haemoglobin
HM: herbal medicine
HMP: herbal medicinal product
HPA: herbal product ‘A’
HPB: herbal product ‘B’
HPC: herbal product ‘C’
HP: herbal product
LDL-C: low-density lipoprotein cholesterol
LYM: lymphocyte count
MCH: mean corpuscular haemoglobin
MCHC: mean corpuscular haemoglobin concentration
MCV: mean corpuscular volume
MPV: mean platelet volume
MRL: maximum residual limit
MXD: mixed monocyte, basophil and eosinophil
OECD: Organization for Economic Cooperation and Development
PDW: platelet distribution width
P-LCR: platelet larger cell ratio
PLT: platelet count
RBC: red blood cell
TG: triglyceride
UCCIRB: University of Cape Coast Institutional Review Board
VLDL-C: very LDL-C
WBC: white blood cell
WHO: World Health Organization
