# Correction: Sub-chronic toxicity evaluation of top-three commercial herbal anti-malarial preparations in the Kumasi metropolis, Ghana

**DOI:** 10.1042/BSR-20192536_COR

**Published:** 2020-07-03

**Keywords:** biochemical, haemolysis, histology, inflammation, safety, Surveillance

In the article “Sub-chronic toxicity evaluation of top-three commercial herbal anti-malarial preparations in the Kumasi metropolis, Ghana” by Adusei-Mensah, F., Tikkanen-Kaukanen, C., Kauhanen, J., Henneh, I.T., Owusu Agyei, P.E., Akakpo, P.K., Ekor M. (*Bioscience Reports* **40**, BSR20192536, DOI: 10.1042/BSR20192536), there was an inadvertent duplication of two of the panels in Figure 5. The histology pictures (photomicrographs), H and Ab, of rats treated with HPC(1) were repeated by mistake as G and Aa for rats that received HPB(10) respectively. The histopathology reports for the images are correct as presented in the result and discussion sections of the article and do not require any changes. The two photomicrographs that were omitted are now correctly inserted in the new Figure 5 below. While this corrected Figure 5 supersedes the figure published in the full article, the overall findings and conclusions of the article remain valid and unchanged.

**Figure 5:**
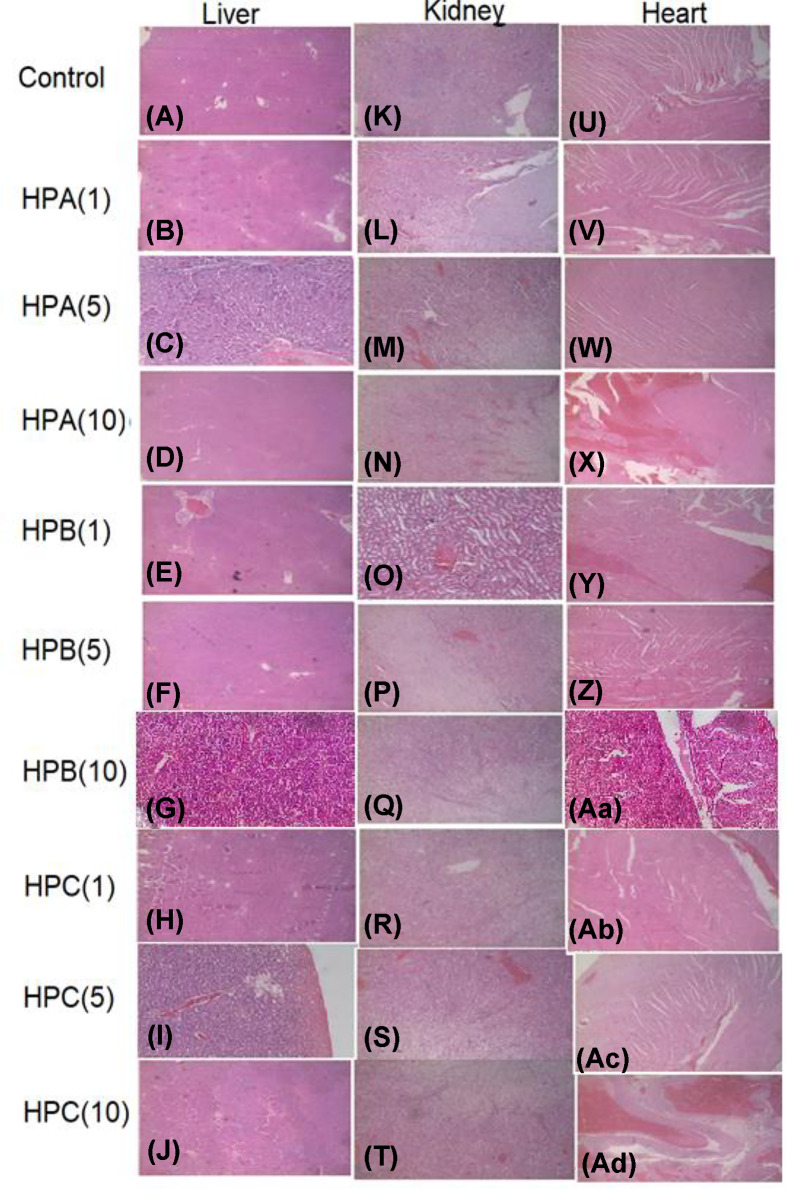
Photomicrographs of the liver, kidney and heart for the sub-chronic toxicity studies of Sprague–Dawley rats treated with either sterile water, HPA, HPB or HPC for 30 days Magnification, ×100. Haematoxylin–Eosin stain was used. Photomicrographs of sections of (**A-J**): liver, (**K-T**): kidney, (**U-Z**) and (**Aa-Ad**): heart.

