# Supplementary material for: Sub-chronic toxicity evaluation of top three commercial herbal antimalarial preparations in the Kumasi metropolis, Ghana
Source: Biosci Rep. 2020 Jun 5;40(6):BSR20192536. doi: 10.1042/BSR20192536 (PMC7276653; doi:10.1042/BSR20192536)
Supplement: Supplementary Figure S1-S2 and Tables S1-S4 [file BSR-2019-2536_supp.pdf]

SUPPLEMENTARY FIGURES

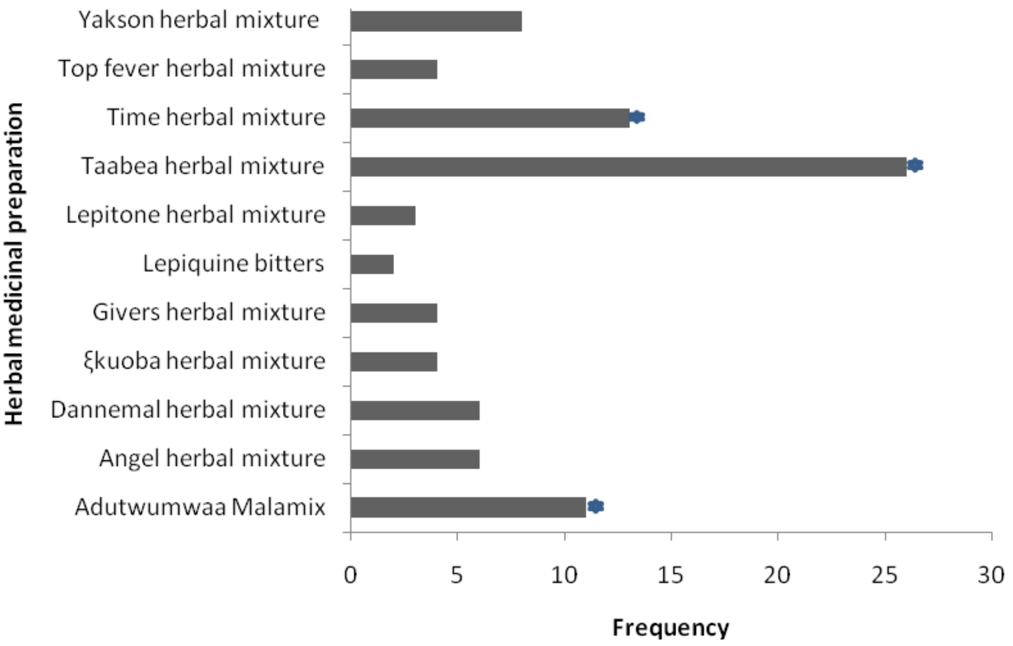

Supplementary Figure 1: Top-three commonly patronized antimalarial herbal medicinal preparations among surveyed participants in the Kumasi metropolis of Ghana[20],[22]. The bars with the star represent the top-three most patronized herbal medicinal products selected for further *in vivo* sub-chronic toxicity study.

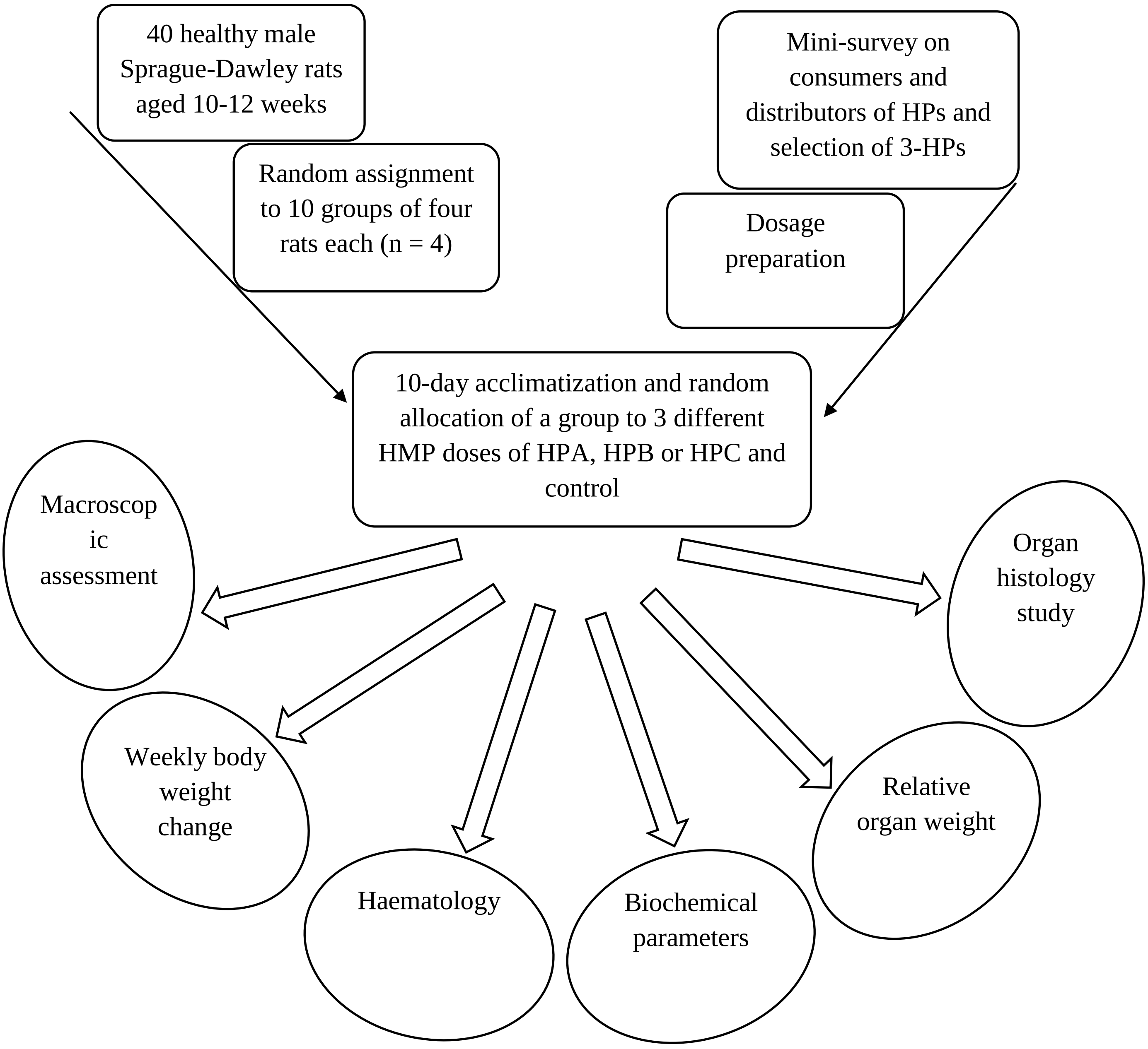
40 healthy male Sprague-Dawley rats aged 10-12 weeks
Random assignment to 10 groups of four rats each (n = 4)
Mini-survey on consumers and distributors of HPs and selection of 3-HPs
Dosage preparation
10-day acclimatization and random allocation of a group to 3 different HMP doses of HPA, HPB or HPC and control
Macroscop ic assessment
Weekly body weight change
Haematology
Biochemical parameters
Relative organ weight
Organ histology study

SupplementaryFigure 2: Study design. The macroscopic, haematological, biochemical, relative organ weights and organ histology studies performed on both test group rats and the control rats.

SUPPLEMENTARY TABLES

SupplementaryTable 1: Ethno-medicinal uses of the medicinal plant constituents

Medicinal plants	Ethnomedicinal uses with reference sources	Documented safety data	Herbal product
Cola gigantea	Stem barks are used for inflammation and bacterial infections. [1] The plant is used in folklore medicine as a heart anti-depressant.	*C. gigantea*oil extract is believed to possessgeneral cellular toxicity effect due to reactive oxygen species production and oxidative stress.[2]	Time herbal mixture
Solanum torvum	Leaves and the unripe fruits are used to treat tuberculosis,[3] *S. torvum*plant is used to treat diabetes,[4] epilepsy,[5] parasitic infections and to reduce oxidative stress on the liver. [6] Extracts from the aerial parts of the plant also have anticancer properties.[7] The plant is also used in Ghana to treat malaria.[8,9]	Acute administration of *S. torvum* was observed to be safe in broiler chickens. [10] *S. torvum* has the potential in preventing the nephrotoxicity induced by doxorubicin.[11] Aqueous fruit extracts had hypotensive effects and were chronically save in rats. [12]	
Spathodeacam panulata	Used for the treatment of malaria,[13] cancer and for healing of wounds.[14]	Acute administration of ethanolic leaf extract was observed to be save in rats, [15] however chronic administration resulted in loss of weight, sluggish movement and significant but reversible hepatotoxic effect. [16]	
Bombaxbuono pozense	Used to treat sleeping sickness. Ethanol extract of the stem bark is also believed to have antitrypanosomal activities. [17]	Activated spines of *B. buonopozense* is reported to have high biosorption for copper and zinc metals, and may lead to bioconcentration of these metals in *B. buonopozense* plant products. [18]	
Vernoniaamyg dalina	Leaf extract of *V. amygdalina*have been reported to affect multiple stages of Plasmodium life cycle, [19,20] leukemia[21] and prostate cancer. [22] It is also used for hepatoprotection. [23,24]	Reported to have chromosomal aberrations effect. [25] Aqueous leaf extract of the plant were observed to have nutritional, clinical and veterinary relevance with no serious hepatotoxic effects in rats. [26]	
Ocimumviride	Ethanol extract of the essential oils from the aerials parts of *O. viride* has anticancer activity against human colorectal adenocarcinoma cells (COLO 205 cell line). [27]*O viride* extract also possess high anti-microbial properties against *Rhizopusstolonifer*, *Aspergillus* sp, and *Fusarium* sp. [28]	Ethanol extract of aerial parts of *O. viride*showed apoptosis properties inducing cytotoxic effect in human colorectal adenocarcinoma cells (COLO 205 cell line) death. [28]	Taabea herbal mixture
Azadirachtain dica	Different parts of the plant are used to treat malaria [29,30], cancer, ulcer, diabetes [31], dengue fever, [32] chicken pox and dermal complications. [31]	Acute and 28-day subacute toxicity tests with *A. indica* fruit oil showed no significant difference in biochemical and haematological parameters, however, at high doses, signs of testicle, liver and kidneys toxicities were observed in histology slides. [33] Seed oil extract was observed by Gandhi and colleagues[334] to cause dose-dependent toxicity on the lungs and central nervous system in both rats and rabbits. [34] An 8-week study with aqueous leaf suspensions showed multi organ toxicities, tremours and loss of weight in goats and guinea pigs. [35]	
Tetrapleuratet raptera	Used in West Africa for the treatment of malaria, [36,37] diabetes and hypertension, inflammation, [38] ulcer, and for the management of epilepsy and childhood convulsions. [39] Extracts of the plant also has a well-studied anti-molluscicide activities for the control of unwanted mollusc vectors. [40]	Edet and Ikpi[41] showed that aqueous fruit extract caused dose dependent mortality in the catfish fingerlings. At high doses, they also observed erratic swimming and loss of balance in the fingerlings. [42]	
Cymbopogonc itratus	Leaf infusion has been used in folklore medicine to treat fever and malaria, [43,44] inflammatory conditions, [44] antifungal infestations [45] and epilepsy. [46]	Oils of *Cymbopogoncitratus*showed a dose-dependent significant functional toxicities to stomach and liver of the Wistar ratduring 14-day toxicity study at doses higher than 1500 mg/kg body weight, the oil was safe at doses less than 1500 mg/kg body weight. [47]	
Moringaoleife ra	The plant is used for treating malaria, [48] diabetes, cancer, [49] and for the treatment of inflammatory-mediated chronic disorders. [50]	*Moringaoleifera* was observed by Asare and colleagues, [51] to exhibit genotoxic at supra-supplementation levels of 3000 mg/kg body weight in rats, but in the same study, they observed intake levels ≤ 1000 mg/kg body	

		weight to be safe in humans. [51] It has also been reported to be safe with no reported toxicity cases in humans. [52]	
Anthocleistan obilis	The plant is used to treat diverse health conditions.It is employed as antidiabetic, antimalarial, antimicrobial, hypotensive, spasmogenic, anti-obesity, antiulcerogenic, analgesic, anti-inflammatory, antioxidant, antitrypanosomal, anthelmintic and fertility agent. [53]	Acute toxicity study of root bark ethanol extract in mice showed toxic neurologic effect and the LD_{50} at 24 h was 200 mg/kg. In the same study, the oral administration of 67 mg/kg ethanol extract attenuated hepatotoxicity in mice induced by CCl4. [54]Not much data is available concerning safety toxicity of the plant in humans. [55]	Adutwumwa amalamix
Vitexgrandifol ia	The bark of the tree is employedfor stomachic purposes and to treat diarrhoea, bronchial complaints, rickets, sores and fever. [56] Also used against malaria, yellow fever, filarial and dengue vector control due to its larvicidal activity. [57] In traditional medicine, the leaves of *V. grandifolia* are used to treat diabetes mellitus and as a diuretic in the treatment of high blood pressure. [58]	Prolonged exposure of *V. grandifolia* is reported to have toxic effects in Sprague-Dawley albino rats, [58]. Observed signs include significant alterations in the architecture of the liver, kidney and lungs in the treated groups compared with the control, significant increase in the serum electrolytes, creatinine, and liver function enzymes in the dosed dependent manner. In addition, signs including polydipsia, polyuria, puffiness of hair, and calmness were reported in rats. [59]	
Phyllanthusfra ternus	Used in Ayurveda and Siddha medicine for the treatment of jaundice and possible anti-DNA polymerase activity of the hepatitis virus. [60] The aerial parts of this plant is also believed to have anti-hepatotoxic activity. [62,62]	Hepatoprotective and antioxidant property of the aqueous extract of *P. fraternus*observed by Lata and colleagues on mice previously administered with cyclophosmide. They observed normalizing of pathological and antioxidant parameters of the cyclophosmide poisoned mice after *P. fraternus* administration. [63]	

1. Agyare C, Koffuor GA, Boamah VE, Adu F, Mensah KB, Adu-Amoah L. Antimicrobial and Anti-Inflammatory Activities of Pterygota macrocarpa and Cola gigantea (Sterculiaceae). *Evid Based Complement Alternat Med*. 2012;2012. doi:10.1155/2012/902394
2. Atolani O, Oguntoye H, Areh ET, Adeyemi OS, Kambizi L. Chemical composition, anti-toxoplasma, cytotoxicity, antioxidant, and anti-inflammatory potentials of Cola gigantea seed oil. *Pharm Biol*. 2019;57(1):154-160. doi:10.1080/13880209.2019.1577468
3. Nguta JM, Appiah-Opong R, Nyarko AK, Yeboah-Manu D, Addo PGA. Medicinal plants used to treat TB in Ghana. *Int J Mycobacteriol*. 2015;4(2):116-123. doi:10.1016/j.ijmyco.2015.02.003
4. Gandhi GR, Ignacimuthu S, Paulraj MG, Sasikumar P. Antihyperglycemic activity and antidiabetic effect of methyl caffeate isolated from Solanum torvum Swartz. fruit in streptozotocin induced diabetic rats. *Eur J Pharmacol*. 2011;670(2-3):623-631. doi:10.1016/j.ejphar.2011.09.159
5. Challal S, Buenafe OEM, Queiroz EF, et al. Zebrafish bioassay-guided microfractionation identifies anticonvulsant steroid glycosides from the Philippine medicinal plant Solanum torvum. *ACS Chem Neurosci*. 2014;5(10):993-1004. doi:10.1021/cn5001342
6. Ramamurthy CH, Subastri A, Suyavaran A, Subbaiah KCV, Valluru L, Thirunavukkarasu C. Solanum torvum Swartz. fruit attenuates cadmium-induced liver and kidney damage through modulation of oxidative stress and glycosylation. *Environ Sci Pollut Res Int*. 2016;23(8):7919-7929. doi:10.1007/s11356-016-6044-3
7. Lu Y, Luo J, Huang X, Kong L. Four new steroidal glycosides from Solanum torvum and their cytotoxic activities. *Steroids*. 2009;74(1):95-101. doi:10.1016/j.steroids.2008.09.011
8. Asase A, Akwetey GA, Achel DG. Ethnopharmacological use of herbal remedies for the treatment of malaria in the Dangme West District of Ghana. *J Ethnopharmacol*. 2010;129(3):367-376. doi:10.1016/j.jep.2010.04.001

9. Abdul Rahuman A, Gopalakrishnan G, Venkatesan P, Geetha K. Isolation and identification of mosquito larvicidal compound from Abutilon indicum (Linn.) Sweet. *Parasitol Res*. 2008;102(5):981-988. doi:10.1007/s00436-007-0864-5
10. Hashemi SR, Zulkifli I, Hair Bejo M, Farida A, Somchit MN. Acute toxicity study and phytochemical screening of selected herbal aqueous extract in broiler chickens. *Int J Pharmacol*. 2008;4(5):352-360.
11. Mohan M, Kamble S, Gadhi P, Kasture S. Protective effect of Solanum torvum on doxorubicin-induced nephrotoxicity in rats. *Food Chem Toxicol*. 2010;48(1):436-440. doi:10.1016/j.fct.2009.10.042
12. Nguelefack TB, Mekhfi H, Dongmo AB, et al. Hypertensive effects of oral administration of the aqueous extract of Solanum torvum fruits in l-NAME treated rats: Evidence from in vivo and in vitro studies. *Journal of Ethnopharmacology*. 2009;124(3):592-599. doi:10.1016/j.jep.2009.04.057
13. Makinde JM, Amusan OO, Adesogan EK. The antimalarial activity of Spathodea campanulata stem bark extract on Plasmodium berghei berghei in mice. *Planta Med*. 1988;54(2):122-125. doi:10.1055/s-2006-962367
14. Agyare C, Asase A, Lechtenberg M, Niehues M, Deters A, Hensel A. An ethnopharmacological survey and in vitro confirmation of ethnopharmacological use of medicinal plants used for wound healing in Bosomtwi-Atwima-Kwanwoma area, Ghana. *J Ethnopharmacol*. 2009;125(3):393-403. doi:10.1016/j.jep.2009.07.024
15. Akharaiyi FC, Boboye B, Adetuyi FC. Study of Acute and Sub Chronic Toxicity of Spathodea campanulata P Beav Leaf. *IPCBEE*. 2012;41.
16. Ilodigwe EE, Akah PA, Nworu CS. Evaluation of the Acute and Subchronic Toxicities of Ethanol Leaf Extract of Spathodea campanulata P. Beauv. *International Journal of Applied Research in Natural Products*. 2010;3(2):17-21.
17. Mann A, Ifarajimi OR, Adewoye AT, et al. In vivo antitrypanosomal effects of some ethnomedicinal plants from Nupeland of north central Nigeria. *Afr J Tradit Complement Altern Med*. 2011;8(1):15-21.
18. Mustapha S, Dauda BEN, Iyaka YA, Mathew TJ, Aliyu IA, Shaba EY. Removal of Heavy Metals from Aqueous Solutions by Modified Activated Carbon from Bombax buonopozense. *IJESI*. 2014;3(8):17-24.
19. Abay SM, Lucantoni L, Dahiya N, et al. Plasmodium transmission blocking activities of Vernonia amygdalina extracts and isolated compounds. *Malar J*. 2015;14:288. doi:10.1186/s12936-015-0812-2
20. Omoregie ES, Pal A. Antiplasmodial, antioxidant and immunomodulatory activities of ethanol extract of Vernonia amygdalina del. Leaf in Swiss mice. *Avicenna J Phytomed*. 2016;6(2):236-247.
21. Yedjou CG, Sims JN, Njiki S, Tsabang N, Ogungbe IV, Tchounwou PB. VERNONIA AMYGDALINA DELILE EXHIBITS A POTENTIAL FOR THE TREATMENT OF ACUTE PROMYELOCYTIC LEUKEMIA. *Glob J Adv Eng Technol Sci*. 2018;5(8):1-9. doi:10.5281/zenodo.1343591
22. Johnson W, Tchounwou PB, Yedjou CG. Therapeutic Mechanisms of Vernonia amygdalina Delile in the Treatment of Prostate Cancer. *Molecules*. 2017;22(10). doi:10.3390/molecules22101594
23. Adesanoye OA, Farombi EO. Hepatoprotective effects of Vernonia amygdalina (astereaceae) in rats treated with carbon tetrachloride. *Exp Toxicol Pathol*. 2010;62(2):197-206. doi:10.1016/j.etp.2009.05.008
24. Imafidon CE, Olukiran OS, Ogundipe DJ, Eluwole AO, Adekunle IA, Oke GO. Acetonic extract of Vernonia amygdalina (Del.) attenuates Cd-induced liver injury: Potential application in adjuvant heavy metal therapy. *Toxicol Rep*. 2018;5:324-332. doi:10.1016/j.toxrep.2018.02.009

25. Okwuzu JO, Odeiga P, AdetoroOtubanjo O, Ezechi OC. Cytotoxicity testing of aqueous extract of bitter leaf (Vernonia amygdalina Del) and sniper 1000EC (2,3 dichlorovinyl dimethyl phosphate) using the Alium cepa test. *Afr Health Sci*. 2017;17(1):147-153. doi:10.4314/ahs.v17i1.19
26. Ojiako OA, Nwanjo HU. Is *Vernonia amygdalina* hepatotoxic or hepatoprotective? Response from biochemical and toxicity studies in rats. *African Journal of Biotechnology*. 2006;5(18). doi:10.4314/ajb.v5i18.55812
27. Sharma M, Agrawal SK, Sharma PR, Chadha BS, Khosla MK, Saxena AK. Cytotoxic and apoptotic activity of essential oil from Ocimum viride towards COLO 205 cells. *Food and Chemical Toxicology*. 2010;48(1):336-344. doi:10.1016/j.fct.2009.10.021
28. Ihejirika GO. Determination of anti-microbial properties of Ocimum viride concentrations on Rhizopus stolonifer infection and germination of soybean (Glycine max L. Merill). *Archives of Phytopathology and Plant Protection*. 2011;44(19):1894-1900. doi:10.1080/03235408.2010.505798
29. Bedri S, Khalil EA, Khalid SA, et al. Azadirachta indica ethanolic extract protects neurons from apoptosis and mitigates brain swelling in experimental cerebral malaria. *Malar J*. 2013;12:298. doi:10.1186/1475-2875-12-298
30. Lucantoni L, Yerbanga RS, Lupidi G, Pasqualini L, Esposito F, Habluetzel A. Transmission blocking activity of a standardized neem (Azadirachta indica) seed extract on the rodent malaria parasite Plasmodium berghei in its vector Anopheles stephensi. *Malar J*. 2010;9:66. doi:10.1186/1475-2875-9-66
31. Saleem S, Muhammad G, Hussain MA, Bukhari SNA. A comprehensive review of phytochemical profile, bioactives for pharmaceuticals, and pharmacological attributes of Azadirachta indica. *Phytother Res*. 2018;32(7):1241-1272. doi:10.1002/ptr.6076
32. Gupta SC, Prasad S, Tyagi AK, Kunnumakkara AB, Aggarwal BB. Neem (Azadirachta indica): An indian traditional panacea with modern molecular basis. *Phytomedicine*. 2017;34:14-20. doi:10.1016/j.phymed.2017.07.001
33. Deng Y, Cao M, Shi D, et al. Toxicological evaluation of neem (Azadirachta indica) oil: Acute and subacute toxicity. *Environmental Toxicology and Pharmacology*. 2013;35(2):240-246. doi:10.1016/j.etap.2012.12.015
34. Gandhi M, Lal R, Sankaranarayanan A, Banerjee CK, Sharma PL. Acute toxicity study of the oil from Azadirachta indica seed (neem oil). *Journal of Ethnopharmacology*. 1988;23(1):39-51. doi:10.1016/0378-8741(88)90113-4
35. Bh A. The toxicity of Azadirachta indica leaves in goats and guinea pigs. *Vet Hum Toxicol*. 1987;29(1):16-19.
36. Okokon JE, Udokpoh AE, Antia BS. Antimalaria activity of ethanolic extract of Tetrapleura tetraptera fruit. *J Ethnopharmacol*. 2007;111(3):537-540. doi:10.1016/j.jep.2006.12.030
37. Lekana-Douki JB, Oyegue Liabagui SL, Bongui JB, Zatra R, Lebibi J, Toure-Ndouo FS. In vitro antiplasmodial activity of crude extracts of Tetrapleura tetraptera and Copaifera religiosa. *BMC Res Notes*. 2011;4:506. doi:10.1186/1756-0500-4-506
38. Ojewole JAO, Adewunmi CO. Anti-inflammatory and hypoglycaemic effects of Tetrapleura tetraptera (Taub) [Fabaceae] fruit aqueous extract in rats. *J Ethnopharmacol*. 2004;95(2-3):177-182. doi:10.1016/j.jep.2004.06.026
39. Ojewole JAO. Analgesic and anticonvulsant properties of Tetrapleura tetraptera (Taub) (Fabaceae) fruit aqueous extract in mice. *Phytother Res*. 2005;19(12):1023-1029. doi:10.1002/ptr.1779

40. Awe SO, Adewunmi CO, Iranloye TA, Ojewole JAO, Olubunmi PA, Becker W. Toxicological evaluation of Aridan, tetrapleura tetraptera (Mimosaceae), a molluscicide. *Toxicological & Environmental Chemistry*. 1995;51(1-4):61-68. doi:10.1080/02772249509358226
41. Edet DI, Ikpi GU. Toxicity and Behaviour of *Clarias Gariepinus* (Burchell, 1822) Fingerlings subjected to Piscicidal Plant Extract of Aidon *Tetrapleura Tetraptera*. *Journal of Applied Sciences and Environmental Management*. 2008;12(3). doi:10.4314/jasem.v12i3.55486
42. Chukwuocha UM, Fernández-Rivera O, Legorreta-Herrera M. Exploring the antimalarial potential of whole Cymbopogon citratus plant therapy. *J Ethnopharmacol*. 2016;193:517-523. doi:10.1016/j.jep.2016.09.056
43. Dike IP, Obembe OO, Adebiyi FE. Ethnobotanical survey for potential anti-malarial plants in south-western Nigeria. *J Ethnopharmacol*. 2012;144(3):618-626. doi:10.1016/j.jep.2012.10.002
44. Francisco V, Costa G, Figueirinha A, et al. Anti-inflammatory activity of Cymbopogon citratus leaves infusion via proteasome and nuclear factor-κB pathway inhibition: contribution of chlorogenic acid. *J Ethnopharmacol*. 2013;148(1):126-134. doi:10.1016/j.jep.2013.03.077
45. Boukhatem MN, Ferhat MA, Kameli A, Saidi F, Kebir HT. Lemon grass (Cymbopogon citratus) essential oil as a potent anti-inflammatory and antifungal drugs. *Libyan J Med*. 2014;9(1):25431. doi:10.3402/ljm.v9.25431
46. Silva MR, Ximenes RM, da Costa JGM, Leal LKAM, de Lopes AA, Viana GS de B. Comparative anticonvulsant activities of the essential oils (EOs) from Cymbopogon winterianus Jowitt and Cymbopogon citratus (DC) Stapf. in mice. *Naunyn Schmiedebergs Arch Pharmacol*. 2010;381(5):415-426. doi:10.1007/s00210-010-0494-9
47. Fandohan P, Gnonlonfin B, Laleye A, Gbenou JD, Darboux R, Moudachirou M. Toxicity and gastric tolerance of essential oils from Cymbopogon citratus, Ocimum gratissimum and Ocimum basilicum in Wistar rats. *Food and Chemical Toxicology*. 2008;46(7):2493-2497. doi:10.1016/j.fct.2008.04.006
48. Prabhu K, Murugan K, Nareshkumar A, Ramasubramanian N, Bragadeeswaran S. Larvicidal and repellent potential of Moringa oleifera against malarial vector, Anopheles stephensi Liston (Insecta: Diptera: Culicidae). *Asian Pac J Trop Biomed*. 2011;1(2):124-129. doi:10.1016/S2221-1691(11)60009-9
49. Adebayo IA, Arsad H, Samian MR. ANTIPROLIFERATIVE EFFECT ON BREAST CANCER (MCF7) OF MORINGA OLEIFERA SEED EXTRACTS. *Afr J Tradit Complement Altern Med*. 2017;14(2):282-287. doi:10.21010/ajtcam.v14i2.30
50. Jaja-Chimedza A, Graf BL, Simmler C, et al. Biochemical characterization and anti-inflammatory properties of an isothiocyanate-enriched moringa (Moringa oleifera) seed extract. *PLoS ONE*. 2017;12(8):e0182658. doi:10.1371/journal.pone.0182658
51. Asare GA, Gyan B, Bugyei K, et al. Toxicity potentials of the nutraceutical Moringa oleifera at supra-supplementation levels. *Journal of Ethnopharmacology*. 2012;139(1):265-272. doi:10.1016/j.jep.2011.11.009
52. Stohs SJ, Hartman MJ. Review of the Safety and Efficacy of Moringa oleifera. *Phytother Res*. 2015;29(6):796-804. doi:10.1002/ptr.5325
53. Anyanwu GO, Nisar-ur-Rehman null, Onyeneke CE, Rauf K. Medicinal plants of the genus Anthocleista--A review of their ethnobotany, phytochemistry and pharmacology. *J Ethnopharmacol*. 2015;175:648-667. doi:10.1016/j.jep.2015.09.032
54. Madubunyi II, Asuzu IU. Pharmacological Screening of Anthocleista nobilis Root Bark. *International Journal of Pharmacognosy*. 1996;34(1):28-33. doi:10.1076/phbi.34.1.28.13175

55. Ngwoke KG, Akwagbulam AG, Erhirhie EO, Ajaghaku DL, Okoye FBC, Esimone CO. Antioxidant, Anti-inflammatory, Analgesic Properties, and Phytochemical Characterization of Stem Bark Extract and Fractions of Anthocleista nobilis. *Pharmacognosy Res*. 2018;10(1):81-87. doi:10.4103/pr.pr_73_17
56. Vitex grandifolia - Useful Tropical Plants. http://tropical.theferns.info/viewtropical.php?id=Vitex+grandifolia. Accessed April 18, 2018.
57. Azokou A, Koné MW, Koudou BG, Tra Bi HF. Larvicidal potential of some plants from West Africa against Culex quinquefasciatus (Say) and Anopheles gambiae Giles (Diptera: Culicidae). *J Vector Borne Dis*. 2013;50(2):103-110.
58. Owolabi MA, Abass MM, Emeka PM, Jaja SI, Nnoli M, Dosa BOS. Biochemical and histologic changes in rats after prolonged administration of the crude aqueous extract of the leaves of Vitex grandifolia. *Pharmacognosy Res*. 2010;2(5):273-278. doi:10.4103/0974-8490.72322
59. Owolabi MA, Abass MM, Emeka PM, Jaja SI, Nnoli M, Dosa BOS. Biochemical and histologic changes in rats after prolonged administration of the crude aqueous extract of the leaves of Vitex grandifolia. *Pharmacognosy Res*. 2010;2(5):273-278. doi:10.4103/0974-8490.72322
60. Rajasubramaniam S, Saradhi PP. Rapid multiplication of Phyllanthus fraternus: a plant with anti-hepatitis viral activity. *Industrial Crops and Products*. 1997;6(1):35-40. doi:10.1016/S0926-6690(96)00201-4
61. Sailaja R, Setty OH. Protective effect of Phyllanthus fraternus against allyl alcohol-induced oxidative stress in liver mitochondria. *J Ethnopharmacol*. 2006;105(1-2):201-209. doi:10.1016/j.jep.2005.10.019
62. Gopi S, Setty OH. Protective effect of Phyllanthus fraternus against bromobenzene induced mitochondrial dysfunction in rat liver mitochondria. *Food Chem Toxicol*. 2010;48(8-9):2170-2175. doi:10.1016/j.fct.2010.05.024
63. Lata S, Singh S, NathTiwari K, Upadhyay R. Evaluation of the Antioxidant and Hepatoprotective Effect of Phyllanthus fraternus Against a Chemotherapeutic Drug Cyclophosphamide. *Appl Biochem Biotechnol*. 2014;173(8):2163-2173. doi:10.1007/s12010-014-1018-8

SupplementaryTable 2: Dosage forms used in this study compared to daily adult human doses (DAHD)

Herbal product	Given code	Dosage in test animals (mg/kg body weight)	Dosage in test animals compared to DAHD (daily dosage for 70 kg man)
Control group	Control.	Normal saline	0
Herbal product 1	HPA(1)	469.98	1x
	HPA(5)	2349.91	5x
	HPA(10)	4699.80	10 x
Herbal product 2	HPB(1)	399.96	1 x
	HPB(5)	1999.80	5 x
	HPB(10)	3999.60	10 x
Herbal product 3	HPC(1)	774.00	1 x
	HPC(5)	3870.00	5 x
	HPC(10)	7740.00	10 x

HPA(1) is the least dose of herbal preparation 'A' and equivalent to DAHD indicated, 5 times HPA(5) and 10 times HPA(10) is the middle and highest doses for herbal preparation 'A' respectively. It was repeated for herbal products 'B' and 'C' with respect to their corresponding DAHD as indicated

SupplementaryTable 3: *Basic information of the herbal product*.

Drug number	Name of Product	Conc. (mg/5ml±sem)	Normal DAHD (ml/70kg/day)	Normal DAHD (mg/kg/day)	Indications
1	Taabea Herbal Mixture	26.11±1.55	90	469.98	malaria, loss of appetite
2	Time Herbal Mixture	22.22±2.66	90	399.96	malaria, loss of appetite, general body pains
3	Adutwumwaa Malamix	43.00±1.16	90	774.00	Malaria

Determined concentrations in 5mL of each preparation; their daily adult human dose (DAHD) (ml/70kg/day), their determined concentrations for the daily human doses (mg/kg/day), their major constituents and their indications on the label.

SupplementaryTable 4: Summary of weekly weight change and haematology results

**Parameter**	HPA(1), HPA(5) and HPA(10)	HPB(1), HPB(5) and HPB(10)	HPC(1), HPC(5) and HPC(10)
BWC W1	NSD	NSD	NSD
BWC W2	NSD	NSD	NSD
BWC W3	NSD	NSD	NSD
BWC W4	NSD	NSD	NSD
HCT (%)	NSD	NSD	NSD
MCV (fL)	NSD	NSD	NSD
MCH (pg)	NSD	NSD	NSD
MCHC (g/dL)	NSD	NSD	NSD
Platelet (x10^3/µL)	NSD	NSD	NSD
Lymphocytes (%)	NSD	NSD	NSD
MXD (%)	NSD	NSD	NSD
Neutrophils (%)	NSD	NSD	NSD
LYM #(x 10^3)	NSD	NSD	NSD
MXD #(x 10^3)	NSD	NSD	NSD
NEUT #(x 10^3)	NSD	NSD	NSD
RDW_SD (fL)	NSD	NSD	NSD
RDW_CV (fL)	NSD	NSD	NSD
PDW (fL)	NSD	NSD	NSD

'NSD' represents no significant difference between the control group and the dosed group at 95% CI. Key: BWC W represents body weight change for weeks (1, 2, 3 and 4). Red blood cell count (RBC), white blood cell count (WBC), granulocyte count (GRA), lymphocyte count (LYM), haemoglobin (HGB), hematocrit (HCT), mean corpuscular haemoglobin (MCH), mean corpuscular volume (MCV), mean corpuscular hemoglobin concentration (MCHC), platelet count (PLT), platelet distribution width (PDW), mean platelet volume (MPV) and platelet larger cell ratio (P-LCR), Mixed cell count (MXD) consisting of *monocytes, eosinophils, basophil.*
